# Prevalence of sleep disturbance and anxiety due to the COVID-19 pandemic in Saudi Arabia

**DOI:** 10.5935/1984-0063.20200079

**Published:** 2021

**Authors:** Shazia Iqbal, Reem Fahad Alanazi, Ali Hussain Alahmed, Anwar Faleh Alnakhli, Mohammed Hejji Alghanim, Manal Abdullah Ali Alghamdi, Shahzad Ahmad

**Affiliations:** 1 Alfarabi College of Medicine, Medical Education Unit - Riyadh - Alfarabi - Saudi Arabia.; 2 FMH College of Medicine and Dentistry, Dentistry Department - Lahore - Pakistan - Pakistan.

**Keywords:** Mental Health, Coronavirus Infections, Anxiety Disorders, Pandemics, Psychological Distress, Sleep Deprivation

## Abstract

**Introduction:**

In the COVID-19 pandemic, physical and psychological health are of immense concern for the governing bodies and health policymakers in the period of lockdown and self-isolation. An in-depth analysis is required to recognize the changes in mental health among the public of different geographical areas.

**Objective:**

The study aimed to investigate the sleep quality and anxiety among the population in Saudi Arabia during the lockdown period from March to June 2020.

**Material and Methods:**

We conducted a cross-sectional study and surveyed the population in Saudi Arabia during the lockdown. We analyzed the anxiety and sleep quality in a population with variable socio-demographic profiles. We assessed anxiety using the self-rating anxiety scale (SAS) questionnaire and tested the sleep quality by using the Pittsburgh sleep quality index (PSQI) questionnaire. We analysed the questionnaire responses to determine the relationships between anxiety, stress, sleep disturbances by using SPSS, and considered the p-value<0.05 statistically significant.

**Results:**

We collected 397 questionnaires from the participants. The respondents were mostly of youth age (19-24 years), 66.5% of respondents were male, while there were 33.5% females. Most of the participants did not contact any COVID-19 patients (75.1%, n=298). The finding revealed that most people did not consider associated anxiety as a worrying disorder as the vast majority were normal with only 12.6% had mild-to-moderate anxiety and almost 1% had moderate-to-severe anxiety. While correlating the sleep patterns disturbance in individuals who had contact with COVID-19 patients, there was a significant sleep disturbance. The disturbance of sleep was in having problems falling sleep (p-value=0.024), having bad/horrible dreams (p-value=0.017), feeling cold at sleeping (p-value=0.038), and difficulty staying active during eating or driving (p-value=0.012). There was a significant correlation with anxiety related to the COVID-19 contact and problems affecting the routine work (p-value=0.001).

**Conclusion:**

There is a significant correlation with anxiety related to the contact with COVID-19 positive patients and problems affecting the routine work among operational professionals. The sleep quality is poor due to the stay-at-home order, having a disorganized working schedule, and deprived lifestyle. The awareness regarding the publics’ mental health related to the pandemic needs to be implemented and psychological guidelines ought to be available for the public. Health measures through the promotion of lifestyle modifications, mindful body practices, meditation, and home exercise can reduce stress and improve the quality of sleep.

## INTRODUCTION

The COVID-19 has a significant impact on the physical and mental health of human beings globally. According to the World Health Organization (WHO), symptoms of COVID-19 have different categories; fever, dry cough, and tiredness and a small percentage of patients with severe COVID-19 might have a hyperinflammatory cytokine storm syndrome. On top of COVID-19 clinical worsening to the patients; it has a considerable impact on the psychological health of the general population^1,2^. There is a significant negative impact on mental health among health professionals, doctors, nurses during the COVID-19^3,4^. There is a significant impact of poor quality of sleep, cognition, high level of anxiety, and deprived mental wellbeing^5,6^.

In Saudi Arabia, there had been strict lockdown measures and self-isolate the population to prevent the spread of the infection as guided by the WHO. As COVID-19 is highly contagious, social distancing and self-isolation were the best preventive measure to decrease the risk of infection spread. However, these precautionary procedures have a significant impact on emotional and mental wellbeing such as the development of chronic insomnia^7,8^. These effects can vary from simple mood changes, depression, aggression, minor anxiety to very severe forms of mental issues such as severe depression, bipolar disorder, and suicidal thoughts^9^.

The presence of social boundaries because of the lockdown, severe educational/work-related interference due to COVID-19, and poorer self-reported physical health were significantly associated with sleep disturbance^10,11^. Additionally, lower perceived social support, lower COVID-19-specified support, and younger age were significantly associated with suicidal thoughts^12^. Some individuals with mild illness, suspected cases of infection, and people who had been in close contact with patients or a potentially high-risk environment were isolated at home. The home isolation is a psychological burden, and even the individual does not have clinical symptoms and remains physically well, they often suffer from negative psychological effects. Better sleep quality has a positive influence on the immune system and reduces the chance to get infected. Thus, psychological health and sleep quality are important factors to be investigated during the COVID-19 pandemic.

The current study explores the anxiety level and sleep disturbances due to the COVID-19 pandemic in Saudi Arabia. As the self-isolation and lockdown was a royal order, the public followed the strict implementation rules in Saudi Arabia. These psychological effects are observed mainly during the lockdown and self-isolation at home because of the spread of COVID-19. The finding will help to estimate the mental health burden of the Saudi public during the COVID-19 pandemic. The recommendations will help the public, health professionals, and policymakers to develop strategies to overcome the sleep and anxiety related to pandemics.

## MATERIAL AND METHODS

We designed a cross-sectional study and targeted the population of Saudi Arabia, who was under isolation at home from March to June 2020 during the COVID-19 pandemic. We collected data through a *Google Docs* questionnaire after consent. During these four-month form was available for participants to fill in and data was collected. The study included adult individuals who had isolation for 2 weeks at home, either they were in contact with COVID-19 patients or not. We performed a convenience sampling to survey the participants of this study. We did not include any infected patients who are hospitalized or under the care of physicians because of COVID-19.

For sample size calculation, we used Raosoft (sample size calculator) to generate the sample size of this research. The minimum recommended sample size was 377 with a confidence level of 95%, the margin of error of 5%, and the response of distribution 50%. To compensate for the missing information, incomplete forms, and other negative contributing factors, the authors added around 5% extra hence, the needed sample size was at least 400 participants.

A self-rated questionnaire is considered the most appropriate way to measure anxiety^13^. Cronbach’s alpha for the total scale was 0.81, which is similar to previous reliability values (0.867) reported in the Chinese population. The participants completed a self-reported questionnaire, which comprised three sections. It related the first section to the socio-demographic data for each participant and disease-related information, which contained 10 questions including gender, age, marital status, work, contact with patients, fever, psychological symptoms, psychological history, and COVID-19 symptoms.

The second section was about sleep quality, which comprised of 16 questions. Pittsburgh sleeps quality index (PSQI)^14^. PSQI is a validated questionnaire and an effective instrument used to measure the quality and patterns of sleep in adults. It differentiates “poor” from “good” sleep by measuring seven areas: subjective sleep quality, sleep latency, sleep duration, habitual sleep efficiency, sleep disturbances, use of sleeping medication, and daytime dysfunction over the last month. The client self-rates each of these seven areas of sleep. It bases the scoring of answers on a 0 to 3 scale, where 3 reflects the negative extreme on the Likert scale. Cronbach’s alpha for the total scale was 0.83, which is similar to previous reliability values and the internal consistency of this questionnaire was previously determined to be 0.87.

The third section concerned anxiety which comprised 20 questions with Likert scale answers (a little of the time, sometimes, good part of the time, most of the time). We collected data in Arabic for the convenience of information collection, and we translated it from Arabic to English for data analysis. We utilized the Zung self-rating anxiety scale (SAS) as a method of measuring levels of anxiety in patients who have anxiety-related symptoms^15^. The scale focused on the most common general anxiety disorders; coping with stress typically post-traumatic stress disorders. The Cronbach’s alpha for internal consistency of this questionnaire was 0.8 and previously determined to be 0.803.

We built the questionnaire in a *Google Forms* to make the collection of data easier during lock-down during the COVID-19 pandemic. Medical experts and researchers reviewed the developed questionnaire at the college of medicine Riyadh for validity. We also assessed it for content validity in terms of content, scope, depth, and appropriateness of each item of the questionnaire. We distributed the questionnaire through the *WhatsApp* groups, *Facebook*, and emails. Participants answered the questionnaires anonymously and provided consent to take part in the study. Upon completion of data collection, we downloaded data on an *Excel* sheet and transformed it into the SPSS sheet.

We analyzed the questionnaire responses to deter mine the relationships between anxiety, stress, sleep disturbances by using SPSS, and considered the *p*-value<0.05 statistically significant. We calculated *p*-value with linear regression models with the t-value from a two-sided t-test for cross-sectional analysis.

## RESULTS

We collected 402 questionnaires, but there were some questionnaires with incomplete information. Hence, we excluded five surveys before the analysis. We selected 397 questionnaires for data analysis and measured the prevalence of sleep disorder according to the Pittsburg sleep quality index in people who isolate themselves to prevent the COVID-19. We measured sleep patterns disturbances and anxiety disorders prevalence during the lockdown period caused by COVID-19. **Table 1** presents the socio-demographic characteristics of the participants.

**Table 1:** Socio-demographic of participants.

Charactristics	Category	Frequency	Percent
**Age (years)**	Less than 18	36	9.1
	19-24	161	40.6
	25-29	95	23.9
	30-34	44	11.1
	More than 35	61	15.4
**Gender**	Male	264	66.5
	Female	133	33.5
**Nationality**	Saudi	360	90.7
	Non-Saudi	36	9.1
**Marital status**	Single	253	63.7
	Married	137	34.5
	Divorced	7	1.8
**Occupation status**	Working	94	23.1
	Non-working	293	76.4
**Dealing with patients**	No	298	75.1
	Yes	99	24.9
**Fever**	No	383	96.5
	Yes	14	3.5
**Respiratory symptoms**	No	370	93.2
	Yes	27	6.8

The respondents were between 19-24 years old age (n=161, 40.6%) and 66.5% of respondents were male while there were 33.5% females. Most of the participants did not contact any COVID-19 patients (75.1%, n=298). Table 2 is showing the measure of sleep disorders in people who isolate themselves to prevent the COVID-19 was according to the Pittsburg sleep quality index (PSQI).

**Table 2:** Prevalence of sleep disorder according to the Pittsburg Sleep Quality Index in people who isolate themselves to prevent the COVID-19

Item	Category	Frequency	Percent
After how many minutes you sleep	1.00	51	12.8
	2.00	135	34.0
	3.00	97	24.4
	4.00	114	28.7
How many hours you sleep	Less than 4	21	5.3
	4-5	49	12.3
	6-7	168	42.3
	8-9	109	27.5
	More than 9	50	12.6
Did you have in last week a problem of more than 30 minutes to sleep	Never	121	30.5
	Less than 1	103	25.9
	1-2 times a week	83	20.9
	3 or more times a week	90	22.7
Do you wake up at night	Never	145	36.5
	Less than 1	88	22.2
	1-2 times a week	76	19.1
	3 or more times a week	88	22.2
Breathing problems during sleeping	Never	310	78.1
	Less than 1	56	14.1
	1-2 times a week	19	4.8
	3 or more times a week	12	3.0
Cough at sleeping	Never	310	78.1
	Less than 1	47	11.8
	1-2 times a week	20	5.0
	3 or more times a week	20	5.0
Feeling Cold at Sleeping	Never	218	54.9
	Less than 1	83	20.9
	1-2 times a week	65	16.4
	3 or more times a week	31	7.8
Fever at Sleeping	Never	268	67.5
	Less than 1	60	15.1
	1-2 times a week	42	10.6
	3 or more times a week	27	6.8
Bad dreams	Never	182	45.8
	Less than 1	113	28.5
	1-2 times a week	70	17.6
	3 or more times a week	32	8.1
Drugs for Sleeping	Never	342	86.1
	Less than 1	20	5.0
	1-2 times a week	15	3.8
	3 or more times a week	20	5.0
Difficulty in Wake up During Eating or Driving	Never	284	71.5
	Less than 1	52	13.1
	1-2 times a week	42	10.6
	3 or more times a week	19	4.8
Difficulty in Wake up During Eating or Driving	Never	284	71.5
	Less than 1	52	13.1
	1-2 times a week	42	10.6
	3 or more times a week	19	4.8
Do your problems affect your work	Never	139	35
	Sometimes	174	43.8
	Frequently	53	13.4
	Always	31	7.8
How was Your Sleeping Last Month	Excellent	81	20.4
	Very Good	166	41.8
	Accepted	103	25.9
	Bad	47	11.8

While correlating the sleep patterns disturbance in individuals who had contact with COVID-19 patients, there was a significant sleep disturbance. Table 3 shows the disturbance of sleep to have problems falling asleep (*p*-value=0.024), having bad/horrible dreams (*p*-value=0.017), feeling cold at sleeping (*p*-value=0.038), and difficulty to stay active during eating or driving (*p*-value=0.012). There was a significant correlation between anxiety related to the COVID-19 contact and problems affecting the routine work (*p*-value=0.001).

**Table 3:** Correlation of sleep patterns disturbance while contacting with COVID-19 patients

Sleep patterns and associated factors	Contact/Dealing with COVID-19 patients
	p-value
Do you have in last week a problem of over 30 minutes to sleep	0.024
Do You Wake Up at Night	0.942
Breathing problems during sleeping	0.078
Cough at Sleeping	0.023
Feeling cold at sleeping	0.038
Bad dreams/Horrible dreams	0.017
Pain in your body	0.053
Drugs for sleeping	0.809
Difficulty in the wake up during eating or driving	0.012
Do your problems affect your work	0.001
How was your sleeping last month	0.219

Measuring the anxiety rate of people who isolate themselves at home to prevent the spread of COVID-19 was assessed using SAS. Table 4 showed the main findings and revealed that anxiety to most people was not considered as a worrying disorder as the vast majority were normal with only 12.6% had mild-to-moderate anxiety and almost 1% had moderate-to- severe anxiety. We linked general anxiety to old age (*p*=0.021), but the younger population noticed to be less prone to have anxiety disorders.

**Table 4:** Anxiety and stress disorders prevalence during the lock-down period caused by COVID-19.

No.	Items	A little of the time 1	Sometimes 2	A good part of the time 3	Most of the time 4
1	I feel more nervous and anxious than usual. %	81 20.40	166 41.80	103 25.90	47 11.80
2	I feel afraid for no reason at all. %	140 35.30	198 49.90	44 11.10	44 3.80
3	I get upset easily or feel panicky. %	224 56.40	135 34.00	24 6.00	14 3.50
4	I feel like I am falling apart and going to pieces. %	273 68.80	76 19.10	33 8.30	15 3.80
5	I feel that everything is all right and nothing bad will happen. %	78 19.60	117 29.50	84 21.20	118 29.70
6	My arms and legs shake and tremble. %	312 78.60	64 16.10	15 3.80	6 1.50
7	I am bothered by headaches, neck and back pain. %	221 55.70	96 24.20	39 9.80	41 10.30
8	I feel weak and get tired easily. %	197 49.60	123 31.00	45 11.30	32 8.10
9	I feel calm and can sit still easily. %	50 12.60	98 24.70	107 27.00	142 35.80
10	I can feel my heart beating fast. %	241 60.70	109 27.50	30 7.60	17 4.30
11	I am bothered by dizzy spells. %	290 73.00	71 17.90	21 5.30	15 3.80
12	I have fainting spells or feel like it. %	361 90.90	30 7.60	4 1.00	2 0.50
13	I can breathe in and out easily. %	31 7.80	27 6.80	30 7.60	309 77.80
14	I get feelings of numbness and tingling in my fingers & toes. %	279 70.30	88 22.20	21 5.30	9 2.30
15	I am bothered by stomach aches or indigestion. %	252 63.50	88 22.20	38 9.60	19 4.80
16	I must empty my bladder often. %	223 56.20	102 25.70	44 11.10	28 7.10
17	My hands are usually dry and warm. %	192 48.40	102 25.70	53 13.40	50 12.60
18	My face gets hot and blushes. %	215 54.20	111 28.00	39 9.80	32 8.10
19	I fall asleep easily and get a good night’s rest. %	90 22.70	123 31.00	92 23.20	92 23.20
20	I have nightmares. %	221 55.70	111 28.00	37 9.30	28 7.10

SAS differentiates the scores based on four categories, (20-44, normal), (45-59, mild-to-moderate anxiety), (60-74, moderate-to-severe anxiety), and (75-80, extreme anxiety). Analysis of each participant’s scores reported in Table 5.

**Table 5:** Anxiety level score among Saudi population during the lock-down period

Anxiety level	Frequency	Percentage
Normal	342	86.1
Mild to Moderate Anxiety	50	12.6
Moderate to Severe Anxiety	5	1.3
Extreme Anxiety	0	0

## DISCUSSION

During the COVID-19 pandemic, physical and psychological health are of immense concern for the public, health professionals, governing bodies, and health policymakers during the period of lockdown^16^. The self-isolation from all aspects of life, such as work, socializing, entertainment, and other various activities can negatively affect the population. There are multiple associated factors to affect mental health such as anxiety, panic disorder, insomnia, depressions, low mood, irritability, and emotional distress^17,18^.

The results revealed that the pandemic has a negative impact in terms of sleep disturbances and anxiety among the general population in Saudi Arabia^19^. There was a significant correlation with anxiety and disturbances related to sleep while having contact with the COVID-19 positive patients^20,21^. Among all the reported mental health issues, we found sleep deprivation because of fear of pandemic and these finding are consistent with the previous studies in China^22^. The sleep disturbance could vary from mild to severe sleep deprivation.

Lockdown related to the COVID-19 pandemic had a major impact on the daily lives of people around the world^23^. In terms of sleep quality, the results showed that the population in Saudi Arabia had poor sleep quality and we link this to sleeping habits. Such results are similar to studies done in China and research endorsed in COVID-19-related psychological interventions should be promoted for better mental health^24,25^.

This investigation shows that relaxed school timetables, disorganized work schedules, and more time spent at home have led people to sleep more hours on average. Hence, the poor sleep quality came from the poor lifestyle and the fact that there are no obligations where the individual must stay at home all the time^26^.

Our study found that 11.8% population felt more nervous and anxious than usual. We linked the general anxiety to old age (*p*=0.021), but the younger population noticed to be less prone to have anxiety disorders. Although previous studies have suggested that the population’s level of anxiety increases with the spread of infectious diseases, the current study did not establish this concern in the included participants. The study found that anxiety-related symptoms are obvious in the population who had contact with COVID-19 patients rather than the general population under lockdown^9,22^.

There are many therapeutic options recommended to overcome pandemic related anxiety, insomnia; such as providing passable safety and protective arrangements to reduce the probability of infection. Few studies recommended adaptions of cognitive behavioural therapy that are realistic to contrivance for the changed work schedules and those with severe anxiety disorder^27^. Timely provision of attentiveness for mental health, especially for healthcare workers or a person at risk for the COVID-19, should be prioritized^22,28^. The authors suggested enhancing home exercises for muscle relaxation to reduce anxiety and improve sleep quality during the COVID-19 pandemic^29^.

Based on the study findings, it is imperative to establish a protocol for psychological inter ventions that need to be incorporated by the ministry of health (MOH) fighting the COVID-19 pandemic for the general population^30,31^. There must be national policy and procedures for psychological intervention for the vulnerable population for general anxiety disorder and depression (known psychiatric patients)^32^.

**Figure 1:**
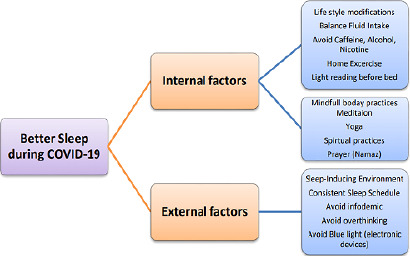
Recommendation to follow the flowchart for boosting sleep and reduce COVID-19 related anxiety.

The health provider must introduce guidelines in the form of leaflets or booklets for the general population to guide them on how to cope with a stressful situation. The authors of the study suggested the flowchart for boosting sleep for the individuals to follow (Figure 1). Health measures through the promotion of lifestyle modifications, mindful body practices, meditation, and home exercise can reduce stress and improve the quality of sleep^33^. There could be organized mental health rehabilitation support for the vulnerable community to enhance mental health^34^. It is a real challenge for health policymakers to design a strateg y for mental health for the general population and establish the results of interventions. The current study is of certain limitations, the study was a cross-sectional study; thus, the results are difficult to be generalized. The participants were recruited using the *WhatsApp* platform, selection bias is highly considered when studying the results. Further research might help to analyze sleep disturbance and pattern of sleep disorders through a mixed-method study design. An in-depth analysis is required to explore the factors associated with the quality of sleep and pattern.

## CONCLUSION

To prevent the spread of COVID-19 pandemics, the Saudi government applies strict rules such as self-isolation at home; the establishment of quarantine institutions, social distancing advertisements, and prohibiting all gathering activities, physical and psychological health are of immense concern for the governing bodies and health policymakers in the period of lockdown and self-isolation. The study was meant to investigate the sleep quality and anxiety among the population in Saudi Arabia during the COVID-19 lockdown period. The study revealed that the general population was not affected in terms of anxiety development, despite the global concern. However, there was a significant correlation with anxiety related to the contact with COVID-19 positive patients and problems affecting the routine work among operational professionals. The sleep quality became poor because of the long stay-at-home, having a disorganized working schedule, and deprived lifestyle.

The awareness regarding the publics’ mental health related to the pandemic needs to be implemented and psychological guidelines ought to be available for the public. Health measures through the promotion of lifestyle modifications, mindful body practices, meditation, and home exercise can reduce stress and improve the quality of sleep. There must be a monitoring of the psychological consequences for outbreaks on health professionals and the establishment of early-targeted mental health interventions.
